# Early Onset Asthma, But Not Aeroallergen Sensitization, Is Associated With Lung Function Impairment in Young Adulthood—A Prospective Cohort Study

**DOI:** 10.1002/clt2.70084

**Published:** 2025-07-23

**Authors:** Joakim Bunne, Linnea Hedman, Anders Bjerg, Matthew Perzanowski, Thomas Platts‐Mills, Eva Rönmark

**Affiliations:** ^1^ Department of Public Health and Clinical Medicine The OLIN and Sunderby Research Unit Umeå University Umeå Sweden; ^2^ Department of Environmental Health Sciences Mailman School of Public Health Columbia University New York New York USA; ^3^ Division of Allergy & Clinical Immunology University of Virginia Charlottesville Virginia USA

**Keywords:** aeroallergen sensitization, asthma, FEV_1_, FVC, prospective study, risk factors

## Abstract

**Background:**

Aeroallergen sensitization is a major factor in asthma, and asthma is associated with impaired lung function. The independent association between sensitization and lung function is unclear.

**Objectives:**

To examine factors associated with lung function in adolescence, with special interests in sensitization and asthma.

**Methods:**

All schoolchildren in grade one and two (median age 8) in two municipalities in Northern Sweden were invited to a questionnaire survey of allergic diseases and skin prick tests to aeroallergens. This was repeated at ages 12 and, at 19 years also including spirometry and *n* = 1495 participated at all three occasions. Associations between risk factors and FEV_1_, FVC and FEV_1_/FVC were analysed by linear regression.

**Results:**

Aeroallergen sensitization was not associated with lung function, irrespective of age at onset, type or degree of sensitization. Early‐onset asthma, both persistent (B −0.35, 95% CI –0.60 to −0.09) and in remission (B −0.43, 95% CI –0.74 to −0.12) was associated with lower FEV_1_. Persistent asthma was associated with lower FEV_1_/FVC (B −0.81, 95% CI –1.07 to −0.55), and remission with lower FVC (B −0.37, 95% CI –0.67 to −0.70). No interaction between asthma and sensitization was found. Maternal smoking in pregnancy was associated with lower FEV_1_/FVC. Underweight at age 19 years was associated with lower FEV_1_ and FVC and overweight was associated with higher FEV_1_ and FVC, but lower FEV_1_/FVC.

**Conclusions:**

Aeroallergen sensitization was not independently associated with lung function. Early onset asthma was strongly associated with lung function impairments in young adulthood and in sensitized and non‐sensitized individuals alike.

**Abbreviations**
BMI: body mass index
FEV_1_
: forced expiratory volume in one second
FEV_1_/FVC: the ratio between FEV_1_ and FVC
FVC: forced vital capacity
ISAAC: International Study of Asthma and Allergy in Children
OLIN: the Obstructive Lung disease In Northern Sweden studies
SPT: skin prick test

## 1 Introduction

Lung function is a marker of health throughout life. Individuals with low lung function in early childhood tend to remain on a trajectory below normal [1]. Likewise, low lung function in young adulthood associates with high prevalence and earlier onset of respiratory and non‐respiratory diseases [2].

Development of lung function is characterized by a rapid growth in childhood with a maximal plateau in young adulthood, and thereafter a slow decrease with age. Several prenatal and early life factors have been linked to impaired lung function development. Maternal smoking during pregnancy increases the risk for respiratory infections and early onset of asthma, which in turn are associated with deterioration in lung function [3, 4, 5]. Small for gestational age and low birthweight have been associated with impaired lung function [6, 7]. Overweight and obesity are associated with impaired lung function in both children and adults, although results are not uniform [8].

It is well known that asthma in childhood associates with subnormal lung function and trajectories thereof [4, 5, 6, 9, 10, 11]. There is also evidence that impaired lung function in infancy predicts asthma in childhood, adolescence and adulthood [12, 13]. Aeroallergen sensitization is a major risk factor for developing asthma [14, 15]^,^, and sensitization also predicts persistence of asthma [16]. The prevalence of aeroallergen sensitization increases with age until young adulthood due to high incidence and low remission, with estimates approaching half of the general population [17, 18]. We recently reported that age at onset of aeroallergen sensitization is an important aspect as early sensitization was associated with very high incidence of asthma and rhinitis, but also with higher levels of specific IgE‐antibodies and poly‐sensitization, compared with late sensitization [19].

Thus, aeroallergen sensitization is an established factor in asthma, which in turn is strongly associated with impaired lung function. Only few studies have investigated the association between sensitization and lung function, with diverging results [4, 9, 20, 21]. However, none of them have taken age at onset of sensitization into account. The aim of the present study was to prospectively investigate factors associated with lung function at 19 years of age, with a special focus on the effects of aeroallergen sensitization and asthma.

## 2 Methods

### 2.1 Study Sample

Within the Obstructive Lung Disease in Northern Sweden (OLIN) research programme, a cohort of schoolchildren was recruited in 1996 by inviting all children in grades 1 and 2 (median age 8 years) in three municipalities in Norrbotten county to a questionnaire survey about allergic diseases. In two of the municipalities, the children were also invited to skin prick testing (SPT) to common airborne allergens [22, 23]. The cohort was followed annually by questionnaire surveys and SPTs were repeated at age 12 years and at 19 years [17, 19]. At 19 years, spirometry for lung function measurements was performed. All investigations took place between February and April, outside the pollen season in this area. The study sample in the current study consisted of the 1495 individuals that participated in the questionnaire survey and SPT at age 8, 12 and 19 years, and the spirometry at 19 years. All studies were approved by the Regional Ethical committee at Umeå University. Written informed consents were obtained from the parents at age 8 and 12 years, and by the individuals them self at age 19 years.

### 2.2 Questionnaire

The questionnaire included the International Study of asthma and Allergies in Childhood (ISAAC) [24] questionnaire with additional questions of symptoms and potential risk factors for allergic conditions [22, 23]. Information on exposures and factors in early life was collected at age 8 years and information on personal smoking, physical activity and body mass index (BMI) was collected at age 19 years (Online Supporting Information S1: Table S1). The questionnaires were answered by parents at age 8 and 12 years, and by the participants at 19 years. The change in respondents was evaluated and the agreement between responses by parents and adolescents was high [25]. At age 8 years, the question of physician‐diagnosis of asthma was validated against clinical assessments with high specificity [23].

### 2.3 Skin Prick Tests

All SPT was carried out according to the European Academy of Allergology and Clinical Immunology (EAACI) guidelines [26] and by a small number of specifically trained personnel [17]. Tested allergens were birch, timothy, and mugwort pollen, cat, dog, horse, two mites (*Dermatophagoides Pteronyssinus* and *Dermatophagoides Farinae*) and two moulds (*Alternaria* and *Cladosporium*). The potency of the extracts was 10 HEP except for the moulds, which were 1:20 w/v. In addition, positive control (histamine 10 mg/mL) and negative control were included. A mean wheal size ≥ 3 mm after 15 min was defined as a positive test. Extracts were provided by ALK, Denmark.

### 2.4 Lung Function

Spirometry was performed with Spirare flow‐volume spirometer (Diagnostica, Oslo, Norway) according to ERS/ATS recommendations [27]. In short, crude values for forced expiratory volume in one second (FEV_1_) and forced vital capacity (FVC) were determined as the best of at least three measurements, and with a repeatability criterion of a difference < 5% or 100 mL between the two best measurements. Bronchodilator response testing was not performed. No specific instructions regarding asthma medications were given. Global Lung Function Initiative (GLI) reference equations [28] were used to calculate predicted values, % of predicted FEV_1_ and FVC, and z‐score for FEV_1_, FVC and FEV_1_/FVC ratio.

### 2.5 Definitions

Sensitization was defined as a positive SPT to any of the tested allergens. Age at onset of sensitization was based on the three time points of SPT in the cohort [19]:

- ≤ 8 years: First time of positive SPT was at age 8 years, that is development of sensitization occurred before age 8 years.
- 8–12 years: First time of positive SPT was at age 12 years, that is development of sensitization occurred between 8 and 12 years.
- 12–19 years: First time of positive SPT was at age 19 years, that is development of sensitization occurred between 12 and 19 years.
- Never: Negative SPT at all ages.

The asthma categories were based upon the presence of current asthma, defined as a physician diagnosis of asthma and in the last 12 months either wheeze or use of asthma medications as reported in the questionnaire.

- Early‐onset persistent: Current asthma at age 8 and 19 years.
- Early‐onset remission: Current asthma at age 8 but not at 19 years.
- Late‐onset: No current asthma at 8 years, but current asthma at either 12 or 19 years.
- Never: No current asthma at any age.
- Further definitions are given in Online Supporting Information S1: Table S1.

### 2.6 Statistical Analyses

Analyses were performed using IBM SPSS Statistics, version 25 IBM Corp. Armonk, NY, NY. The lung function variables FEV_1_, FVC and FEV_1_/FVC are presented as z‐score and % predicted. Students *t*‐test was used for comparisons of means between two groups and ANOVA for comparisons between more than two groups. Chi‐square test was used for comparisons of proportions between groups. Variables in unadjusted analysis that were significantly (*p*‐value < 0.05) or borderline significantly (*p*‐value < 0.15) associated with any lung function outcome at age 19 years were included as covariates in adjusted linear regression analyses. Sensitization was included in all analyses as it was an exposure of special interest. First, only risk factors assessed longitudinally or at 8 years were included, and in next step, factors assessed at 19 years were included. Sensitivity analyses were performed by excluding individuals with asthma, and by replace age at onset of any aeroallergen sensitization with sensitization to any animal and any pollen, respectively, at 19 years, and poly‐sensitization at 8 and 19 years, respectively. Adjusted results are presented as Beta (B) coefficient with 95% confidence intervals (CI). Potential interaction between asthma categories and sensitization was evaluated by separate analyses among sensitized and non‐sensitized individuals.

## 3 Results

### 3.1 Characteristics of Study Sample

Crude values of FEV_1_, FVC and FEV_1_/FVC in the study sample differed by sex, while there were no differences for z‐score or % predicted. Among women, 2.1% had FEV_1_/FVC < LLN, while the corresponding prevalence among men was 3.3% (Table 1).

**TABLE 1:** Forced expiratory volume in one second (FEV_1_), forced vital capacity (FVC) and FEV_1_/FVC at age 19 years in the study sample, by sex and in total.

Lung function, mean (SD)	Females	Males	All	*p*‐value for difference by sex^a^
	*n* = 747	*n* = 748	*n* = 1495	
FEV_1_, litres	3.37 (0.44)	4.62 (0.57)	4.00 (0.81)	< 0.001
FEV_1_, % predicted	97.4 (10.4)	98.3 (10.7)	97.8 (10.6)	0.112
FEV_1_, z‐score	−0.22 (0.89)	−0.14 (0.93)	−0.18 (0.91)	0.133
FVC, litres	3.79 (0.51)	5.38 (0.71)	4.58 (1.00)	< 0.001
FVC, % predicted	96.2 (10.6)	97.0 (10.9)	96.6 (10.8)	0.164
FVC, z‐score	−0.32 (0.87)	−0.27 (0.95)	−0.29 (0.91)	0.263
FEV_1_/FVC	0.89 (0.05)	0.86 (0.06)	0.88 (0.06)	< 0.001
FEV_1_/FVC, z‐score	0.16 (0.90)	0.13 (0.96)	0.14 (0.93)	0.522
FEV_1_/FVC < LLN, %	2.1	3.3	2.7	0.155

^a^ *t*‐test for numerical data, chi square test for proportions.

Sensitization to all common allergens increased by age. Prevalence of sensitization to any allergen at age 19 years was 42.9%, and was more common among men. Sensitization to cat (13.9%) and dog (8.6%) were more common than sensitization to birch (8.2%) and timothy (6.6%) at age 8 years, while at age 19 years these four most common allergens showed similarly high prevalence (23%–28%). Sensitization to mites and moulds was uncommon at all ages (Table 2). In total, 13.8% had current asthma at any occasion, 8.2% had late‐onset, 3.4% early‐onset persistent asthma and 2.3% early‐onset remission (Table 3). The sensitization patterns among the asthma categories reflected the whole sample but the percentage of sensitized individuals were much higher and highest among those with early onset of asthma (Table 2). Early‐onset asthma in remission was more common in men. Regarding factors assessed at 19 years, exercise ≥ 4 times/week was more common among men. Smoking was more common among women, 11.2%, than men, 8.2%. Underweight was twice as common among women, 9.6% versus 4.1%, while the reverse was found for overweight and obesity with a prevalence of 25.9% and 8.8%, respectively among men and 11.1% and 5.1% among women (Online Supporting Information S1: Table S2).

**TABLE 2:** Prevalence (%) of aeroallergen sensitization in the whole study sample by age and sex, and in the different asthma categories.

		In the whole sample				In individuals with asthma		
Allergen	Age	All (*n* = 1495)	Girls (*n* = 747)	Boys (*n* = 748)	*p*‐value^a^	Early‐onset‐persistent (*n* = 51)	Early‐onset‐remission (*n* = 34)	Late‐onset (*n* = 122)
Birch	8 years	8.2	7.1	9.2	0.131	29.4	20.6	19.0
	12 years	14.8	13.1	16.6	0.060	39.2	38.2	32.0
	19 years	23.4	21.3	25.5	0.052	47.1	50.0	41.0
Timothy	8 years	6.6	4.7	8.4	0.004	17.6	18.2	9.1
	12 years	15.5	12.7	18.2	0.003	31.4	35.3	23.0
	19 years	27.7	23.6	31.8	< 0.001	51.0	52.9	46.7
Mugwort	8 years	1.0	0.8	1.2	0.435	6.0	0	0.8
	12 years	3.1	2.8	3.5	0.461	11.8	11.8	4.9
	19 years	6.2	5.2	7.2	0.111	15.7	8.8	12.4
Cat	8 years	13.9	12.5	15.3	0.121	51.0	36.4	32.0
	12 years	20.1	16.9	23.3	0.002	56.9	50.0	47.5
	19 years	27.3	23.7	30.9	0.002	60.8	58.8	49.2
Dog	8 years	8.6	7.7	9.5	0.198	41.2	37.5	18.9
	12 years	18.1	14.9	21.3	0.001	54.9	50.0	41.0
	19 years	27.4	22.9	32.0	< 0.001	62.7	55.9	56.6
Horse	8 years	6.2	5.1	7.4	0.071	37.3	27.3	12.4
	12 years	11.0	9.5	12.6	0.059	45.1	32.4	27.0
	19 years	14.9	13.1	16.7	0.051	43.1	38.2	35.2
Any mite^b^	8 years	1.1	0.3	1.9	0.002	3.9	0.0	2.5
	12 years	1.3	0.8	1.9	0.073	2.0	8.8	0.8
	19 years	3.7	3.2	4.3	0.278	7.8	5.9	5.7
Any mould^c^	8 years	1.4	0.5	2.3	0.04	13.7	3.3	4.1
	12 years	0.9	0.5	1.3	0.108	3.9	2.9	1.6
	19 years	2.4	1.6	3.2	0.043	11.8	5.9	4.9
Any allergen	8 years	21.1	18.9	23.4	0.032	60.8	47.1	40.2
	12 years	30.9	26.9	34.9	< 0.001	66.7	67.6	59.8
	19 years	42.9	39.5	46.4	0.007	80.4	73.5	72.1

^a^ differences by sex.

^b^ any of the allergens Der. Pteronussinus or Der. Farinae.

^c^ any of the allergens Cladosporium or Alternaria.

**TABLE 3:** Prevalence (%) of risk factors in the study sample, and FEV_1_, FVC and FEV_1_/FVC in z‐score with standard deviation (SD) at age 19 years by risk factors.

	Prevalence % (n)	FEV_1_		FVC		FEV_1_/FVC	
Factors assessed at age 8 or longitudinally		z‐score (SD)	*p*‐value^a^	z‐score (SD)	*p*‐value^a^	z‐zcore (SD)	*p*‐value^a^
Age at onset of sensitization							
Never	55.9 (835)	−0.18 (0.89)	0.182	−0.30 (0.90)	0.157	0.16 (0.91)	0.608
> 12–19 years	12.3 (184)	−0.25 (1.06)		−0.39 (0.96)		0.16 (0.91)	
> 8–12 years	10.7 (160)	−0.05 (0.87)		−0.16 (0.91)		0.14 (0.95)	
≤ 8 years	21.1 (316)	−0.22 (0.90)		−0.29 (0.89)		0.08 (0.99)	
Number of positive SPT at 8 years							
0	78.9 (1179)	−0.17 (0.91)	0.157	−0.29 (0.91)	0.694	0.16 (0.91)	0.123
1	9.1 (136)	−0.10 (0.86)		−0.23 (0.89)		0.17 (0.90)	
2	5.1 (76)	−0.28 (0.82)		−0.38 (0.83)		0.12 (1.00)	
3 or more	7.0 (104)	−0.34 (0.98)		−0.32 (0.92)		−0.07 (1.08)	
Asthma category							
Never	86.2 (1288)	−0.16 (0.89)	**0.001**	−0.30 (0.90)	**0.021**	0.19 (0.91)	**<** **0.001**
Late‐onset	8.2 (122)	−0.20 (1.04)		−0.22 (0.98)		−0.03 (0.96)	
Early‐onset‐remission	2.3 (34)	−0.63 (0.88)		−0.68 (0.92)		0.02 (0.81)	
Early‐onset‐persistent	3.4 (51)	−0.50 (0.98)		−0.08 (0.87)		−0.71 (0.90)	
Birthweight < 2500 g							
No		−0.17 (0.90)	0.221	−0.29 (0.90)	0.358	0.14 (0.92)	0.994
Yes	3.8 (55)	−0.33 (0.99)		−0.40 (1.08)		0.14 (1.09)	
Severe respiratory infection							
No		−0.13 (0.89)	0.056	−0.28 (0.91)	0.536	0.21 (0.94)	0.019
Yes	56.7 (848)	−0.22 (0.92)		−0.31 (0.91)		0.09 (0.91)	
Maternal smoking during pregnancy							
No		−0.17 (0.92)	0.369	−0.31 (0.92)	0.070	0.20 (0.93)	< 0.001
Yes	23.0 (340)	−0.22 (0.86)		−0.21 (0.85)		−0.06 (0.89)	
Parental asthma							
No		−0.17 (0.91)	0.301	−0.30 (0.91)	0.491	0.17 (0.91)	0.004
Yes	17.7 (264)	−0.23 (0.92)		−0.26 (0.89)		−0.01 (0.98)	
Breastfeeding < 3 months							
No		−0.18 (0.90)	0.300	−0.29 (0.91)	0.516	0.15 (0.92)	0.492
Yes	13.8 (207)	−0.25 (0.93)		−0.33 (0.90)		0.10 (0.92)	
Traffic pollution exposure							
No		−0.17 (0.92)	0.730	−0.28 (0.92)	0.529	0.13 (0.94)	0.666
Yes	41.5 (621)	−0.19 (0.89)		−0.31 (0.89)		0.15 (0.91)	
Factors assessed at 19 years							
Sensitization to any animal							
No		−0.16 (0.90)	0.259	−0.29 (0.91)	0.957	0.17 (0.89)	0.060
Yes	44.8 (506)	−0.22 (0.94)		−0.29 (0.90)		0.08 (0.98)	
Sensitization to any pollen							
No		−0.18 (0.90)	0.896	−0.30 (0.90)	0.845	0.15 (0.92)	0.778
Yes	34.6 (517)	−0.18 (0.94)		−0.29 (0.92)		0.13 (0.94)	
Number of positive SPT							
0	57.1 (853)	−0.17 (0.89)	0.722	−0.30 (0.90)	0.648	0.17 (0.91)	0.639
1	9.8 (147)	−0.24 (1.01)		−0.35 (0.98)		0.12 (0.87)	
2	6.8 (101)	−0.25 (0.96)		−0.32 (1.01)		0.09 (0.96)	
3 or more	26.4 (394)	−0.16 (0.90)		−0.25 (0.87)		0.10 (0.98)	
Current allergic rhinitis							
No	86.1 (1287)	−0.19 (0.91)	0.358	−0.31 (0.99)	0.164	0.15 (0.93)	0.329
Yes	13.9 (208)	−0.13 (0.88)		−0.21 (0.83)		0.08 (0.93)	
Smoker							
No		−0.17 (0.91)	0.140	−0.39 (0.90)	0.164	0.15 (0.93)	0.615
Yes	9.7 (145)	−0.29 (0.95)		−0.28 (0.91)		0.10 (0.86)	
Exercise ≥ 4 times/week							
No		−0.26 (0.92)	**<** **0.001**	−0.36 (0.91)	**<** **0.001**	0.14 (0.95)	0.791
Yes	39.0 (583)	−0.06 (0.89)		−0.19 (0.90)		0.15 (0.90)	
Body Mass index category^a^							
Underweight	6.9 (103)	−0.73 (0.89)	**<** **0.001**	−1.02 (0.90)	**<** **0.001**	0.55 (1.01)	**<** **0.001**
Normal	67.6 (1010)	−0.17 (0.89)		−0.32 (0.86)		0.21 (0.95)	
Overweight	18.5 (277)	−0.02 (0.86)		−0.01 (0.90)		−0.11 (0.74)	
Obesity	7.0 (104)	−0.14 (1.04)		−0.04 (0.99)		−0.25 (0.75)	

*Note:* Underweight = BMI < 18.5, normal = BMI 18.5–24.9; overweight = BMI 25–29.9, obesity = BMI > 30.

^a^ Significant associations highlighted in bold.

### 3.2 Factors Associated With Lung Function, Unadjusted Analyses

Aeroallergen sensitization, irrespective of age at onset, was not related to any lung function parameter. Those with early‐onset persistent asthma had significantly lower FEV_1_/FVC compared with never asthma, z‐score −0.71 versus. 0.19 (*p* < 0.001). Both early‐onset remission and early‐onset persistent asthma had significantly lower FEV_1_ compared with never asthma, z‐score −0.63 and −0.50 versus −0.10, respectively (Table 3).

Severe respiratory infection in childhood, maternal smoking during pregnancy and parental asthma were all negatively associated with FEV_1_/FVC, but not significantly so with FEV_1_ or FVC. Neither birthweight < 2500 g nor breastfeeding < 3 months was associated with any lung function outcome.

BMI at age 19 years displayed several significant associations with lung function. As compared to normal weight, underweight individuals had lower FEV_1_ (z‐score −0.73 vs. −0.17) and FVC (z‐score −1.02 vs. −0.32), while FEV_1_/FVC was higher. Those with overweight and obesity had higher FVC, and lower FEV_1_/FVC (z‐score −0.11 and −0.25 vs. 0.21, respectively). Personal smoking was not significantly associated with any outcome, while exercise ≥ 4 times/week was associated with higher FEV_1_ and FVC. At age 19 years, sensitization to any animal was borderline significant for decreased FEV_1_/FVC, while no effect was seen for sensitization to pollen, nor for poly‐sensitization. Among those with current allergic rhinitis at age 19 years, 94% were sensitized to any allergen, and no association with lung function was found (Table 3). Results presented as % predicted and crude FEV_1_/FVC are found in Online Supporting Information S1: Table S3.

We further analysed the association between asthma and lung function stratified by aeroallergen sensitization. We found no significant difference in the lung function parameters by sensitization status in any of the asthma categories, or among those never having had asthma (Table 4).

**TABLE 4:** Lung function, FEV_1_, FVC and FEV_1_/FVC, in z‐score with standard deviation (SD) by asthma categories and among individuals ever and never sensitized to airborne allergens, respectively, at age 19 years.

		FEV_1_		FVC		FEV_1_/FVC	
Asthma category	Sensitization status	z‐score (SD)	*p*‐value	z‐score (SD)	*p*‐value	z‐score (SD)	*p*‐value
Never asthma	Never sensitized (*n* = 782)	−0.16 (0.87)	0.770	−0.29 (0.89)	0.634	0.17 (0.91)	0.310
	Ever sensitized (*n* = 506)	−0.15 (0.92)		−0.31 (0.91)		0.23 (0.91)	
Late‐onset asthma	Never sensitized (*n* = 34)	−0.28 (1.21)	0.603	−0.34 (1.12)	0.390	0.05 (0.93)	0.568
	Ever sensitized (*n* = 88)	−0.17 (0.79)		−0.17 (0.92)		−0.06 (0.96)	
Early‐onset‐remission	Never sensitized (*n* = 9)	−0.89 (1.03)	0.316	−0.96 (1.07)	0.294	0.03 (0.69)	0.963
	Ever sensitized (*n* = 25)	−0.54 (0.83)		−0.58 (0.86)		0.01 (0.87)	
Early‐onset‐persistent	Never sensitized (*n* = 10)	−0.37 (0.68)	0.641	−0.17 (0.59)	0.731	−0.45 (0.56)	0.300
	Ever sensitized (*n* = 41)	−0.53 (1.04)		−0.06 (0.93)		−0.78 (0.96)	

*Note: p*‐value by sensitization status.

### 3.3 Adjusted Analyses

In the model only including early life factors and factors collected longitudinally no significant effect was seen for sensitization, regardless of age at onset. Early‐onset persistent asthma was associated with lower z‐scores of FEV_1_ and FEV_1_/FVC. Early‐onset remission was associated with lower FEV_1_ and FVC, and late‐onset asthma had lower FEV_1_/FVC. Maternal smoking during pregnancy was also associated with lower FEV_1_/FVC (Table 5).

**TABLE 5:** Risk factors associated with FEV1, FVC and FEV1/FVC in z‐score at 19 years and analysed by adjusted linear regression.

		FEV_1_, *z* score			FVC, z‐score			FEV_1_/FVC, z‐score		
Risk factors assessed longitudinally or at age 8 years		B	(95% CI)	*p*‐value	B	(95% CI)	*p*‐value	B	(95% CI)	*p*‐value
Age at onset of sensitization	Never	Ref								
	> 12–19 years	−0.053	(−0.200‐0.095)	0.485	−0.098	(−0.245‐0.049)	0.193	0.051	(−0.096–0.199)	0.496
	> 8–12 years	0.152	(−0.002–0.307)	0.054	0.127	(−0.028–0.282)	0.107	0.040	(−0.115–0.195)	0.615
	≤ 8 years	0.006	(−0.117–0.128)	0.929	−0.013	(−0.136‐0.110)	0.836	0.031	(−0.115–0.195)	0.619
Asthma category	Never	Ref								
	Late‐onset	−0.059	(−0.235‐0.117)	0.511	0.037	(−0.139–0.213)	0.680	−0.180	(−0.356 to −0.004)	**0.045**
	Early‐onset‐remission	−0.476	(−0.790 to −0.162)	**0.003**	−0.421	(−0.734‐‐0.107)	**0.009**	−0.118	(−0.432‐0.196)	0.462
	Early‐onset‐persistent	−0.315	(−0.577 to −0.054)	**0.018**	0.214	(−0.047–0.475)	0.108	−0.861	(−1.123 to −0.600)	**<** **0.001**
Severe respiratory infection		−0.072	(−0.166‐0.022)	0.134	−0.038	(−0.132‐0.055)	0.424	−0.066	(−0.160‐ 0.028)	0.167
Maternal smoking during pregnancy		−0.050	(−0.160‐0.060)	0.377	−0.092	(−0.018–0.202)	0.101	−0.242	(−0.352 to −0.132)	**<** **0.001**
Parental asthma		−0.017	(−0.141‐0.107)	0.786	0.046	(−0.078–0.170)	0.465	−0.103	(−0.228‐0.021)	0.104

*Note:* Significant *p*‐values are presented in bold.

**FIGURE 1:**
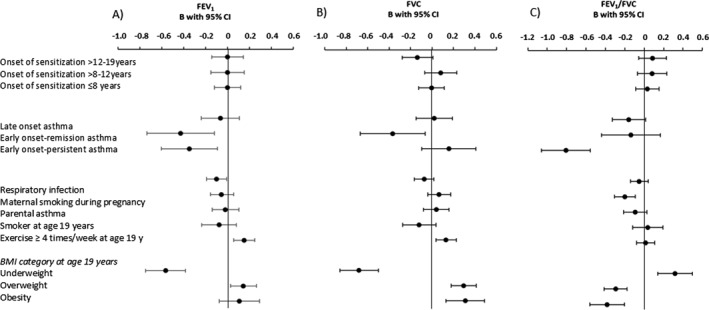
Factors associated with (A) FEV_1_, (B) FVC and (C) FEV_1_/FVC in z‐score at age 19 years, analysed by adjusted linear regression and presented as B‐coefficient with 95% confidence interval. All presented variables were included in the respective model. BMI = body mass index; underweight = BMI < 18.5, overweight = BMI25–29.9, Obesity = BMI > 30. FEV_1_ = forced expiratory volume in one second; FVC = forced vital capacity. Never sensitized was reference category for age at onset of sensitization categories; never asthma was reference for the asthma categories; normal weight was reference for BMI categories.

In the next step we also included factors assessed at 19 years (Figure 1). Still, no significant associations were found between sensitization and lung function. The associations for early‐onset persistent and early‐onset remission remained significant and of similar size, while late‐onset asthma was no longer significantly associated with FEV_1_/FVC. Maternal smoking during pregnancy remained significantly associated with lower FEV_1_/FVC, and respiratory infections became significantly associated with lower FEV_1_. Regarding factors assessed at 19 years, the patterns found in bivariate analysis remained: exercise ≥ 4 times/week was associated with higher FEV_1_ and FVC; underweight was associated with lower FEV_1_ and FVC, and with higher FEV_1_/FVC; overweight was associated with higher FVC and FEV_1_. The same pattern was found for obesity, although not significantly so for FEV_1_. In contrast, both overweight and obesity were associated with lower FEV_1_/FVC. The pattern was similar in analyses stratified by sex, although statistical significance was not reached for some factors.

In sensitivity analysis age at onset of any allergen sensitization was replaced with poly‐sensitization and sensitization to any animal and any pollen, respectively, at age 19 years. No significant effects were found for any of these, and the results for other covariates remained. The effect estimates of included factors did not change in a further analysis restricted to individuals without asthma.

## 4 Discussion

In this longitudinal study among individuals followed from age 8, aeroallergen sensitization was unrelated to lung function at age 19 years. This finding was robust in all adjusted models, as well as in unadjusted analyses. Early‐onset asthma, particularly persistent but also asthma in remission was associated with airway obstruction, defined as lower FEV_1_ or FEV_1_/FVC, and this was observed among sensitized and non‐sensitized individuals alike. Respiratory infections before school age and maternal smoking during pregnancy were both associated with lower lung function at 19 years. Frequent exercise and overweight at age 19 years were associated with higher FEV_1_ and FVC, while the opposite was found for underweight.

### 4.1 Allergic Sensitization and Lung Function

As the association between asthma and impaired lung function is well established and allergen sensitization is a strong risk factor for asthma, we expected sensitization, particularly early onset, to negatively affect lung function. However, we did not find any association between aeroallergen sensitization and lung function. Surprisingly, this was evident already in unadjusted analysis. The adjusted models did not alter the findings, and neither did a sensitivity analysis restricted to individuals without asthma. This is unlikely to be a result of insufficient statistical power, as no trend towards a difference was seen. Previous studies have shown conflicting results on this topic. In the Isle of Wight cohort, positive SPT at age 4 was associated with low lung function trajectories until age 26 years [21]. In the MAAS cohort, sensitization at 3 years was part of a model predicting low lung function trajectory, and poly‐sensitization was associated with impaired lung function until age 11 years [29]. However, no association between sensitization in adolescence (MAAS) or at 7 years (ALSPAC), and low lung function trajectory was found [4]. In the high‐risk COPSAC cohort, sensitization at 13 years did not alter lung function development until that age [30]. In a New Zeeland cohort, sensitization to house dust mite was associated with impaired FEV_1_/FVC at 26 years, but sensitization to any allergen at 13 or 21 years was not [20]. Finally, in the Swedish population‐based BAMSE cohort, sensitization at 8 years was not associated with low FEV_1_ or low FEV_1_ growth between 8 and 16 years [9]. Importantly, these studies did not separate the effects of asthma and sensitization. Thus, an observed association between sensitization and lung function may be due to the association between sensitization and asthma, which in turn impairs lung function [31]. This is supported by our detailed analyses showing no independent effect of aeroallergen sensitization in school age on lung function evaluated in early adulthood.

The prevalence of sensitization and dominance of furred animals and pollen, is in keeping with other Swedish findings [14] but contrasts to populations in warmer climate where e.g. house dust mite is a major sensitizer [18]. In unadjusted analysis, there was a trend towards effect of animal sensitization on FEV_1_/FVC, which however disappeared when adjusting for asthma. Asthma is more strongly associated with allergic sensitization to animals than to pollen [16]. This finding further underlines that there was no independent association of sensitization with lung function, as animal sensitization only more closely identifies individuals at increased risk of developing asthma. Allergic rhinitis is strongly associated with sensitization both to animals and pollen, but allergic rhinitis was in contrast to asthma, not associated with impaired lung function.

### 4.2 Asthma and Lung Function

Early‐onset persistent asthma was robustly associated with airway obstruction in line with several other studies [5, 10, 32, 33]. While individuals with early‐onset persistent asthma had lower FEV_1_and FEV_1_/FVC, i.e. airway obstruction, those with early‐onset asthma in remission showed lower values of both FVC and FEV_1_ yielding a normal FEV_1_/FVC ratio. Thus, as compared with persistent asthma, those in remission had small lungs rather than airway obstruction. However, we must keep in mind that those in remission by definition had not used asthma medications during the last 12 months. No instruction was given to withhold asthma medications prior to spirometry. Thus, those with persistent asthma may have better values due to treatment and our results underestimate the negative impact of asthma. Also, we did not test for bronchodilator response, i.e. reversibility cannot be assessed. Nonetheless, those in remission had similar reduction in FEV_1_ as those with persistent asthma, arguing against this putative medication effect. A possible explanation for the findings among those in remission is underlying inflammatory activity and/or persisting physiological alterations in the airways, despite clinical remission from asthma. This has been shown in long‐term follow up of childhood asthmatics in the Netherlands [34] and childhood asthma with remission in adulthood has been shown to associate with accelerated decline in FEV_1_ and FVC [35]. In contrast to early‐onset asthma, lung function among those with late‐onset did not differ from subjects without asthma. This could be due to shorter time of having asthma, differences in pathophysiology, or both. Also, as with the persistent group, use of treatment might attenuate an effect on lung function. Taken together, there is clear evidence that early‐onset asthma significantly affects lung function in adulthood.

To further investigate a potential interaction between sensitization and asthma, we analysed the effect of asthma on lung function stratified by sensitization status. There was no difference for any of the asthma categories between sensitized and non‐sensitized, and no trend suggesting an effect of sensitization on lung function. Using allergic sensitization as a marker for T2‐high asthma, our results suggest that the effect of T2‐high asthma on lung function at age 19 years does not differ from T2‐low asthma, a finding in line with others [36]. It has been suggested that T2‐low asthma may be more important than previously thought among school‐aged children [37]. The fact that early‐onset and in particularly persistent asthma was associated with worse lung function suggests that features of the disease itself rather than sensitization determine lung function impairment. Of interest, but not within our aim of this study, is that by defining asthma by age at onset, the prevalence of aeroallergen sensitization is not stable but increases by the age at testing for sensitization. This is mainly a result of the high incidence of sensitization in the ages under study but highlight that a phenotype of asthma, e.g. aeroallergen sensitization or not, is dynamic over time [38].

### 4.3 Other Factors and Lung Function

Severe respiratory infection before age 8 was associated with airway obstruction. Although in line with other studies [4, 5], we interpret this finding with caution as it may be prone to recall bias and respiratory infections are more common among those with asthma. However, the association remained after adjustment for asthma suggesting an independent effect. Maternal smoking in pregnancy, although also assessed at age 8, may be less susceptible to such bias and in line with others [4] we found an association with lower FEV_1_/FVC indicating obstruction. Personal smoking was not associated with impaired lung function at age 19 years, probably due to short duration of smoking. However, in the same cohort we have in a previous publication reported that smoking was associated with respiratory symptoms already at age 17 years [39]. Low birth weight was not associated with lung function at 19 years in our study, contrary to other findings [6, 7]. However, the proportion with low birth weight was small, and unfortunately, we are lacking other pregnancy data (e.g. gestational age). Regardless, this reinforces the notion that in utero exposures may significantly affect lung health.

The association between BMI and lung function, both collected at age 19 years, was a significant finding with similar strength among both sexes despite a higher prevalence of underweight among women and of overweight among men. As compared to normal weight, underweight was associated with marked decreases in mean FEV_1_ and FVC indicating small lungs in general but not obstruction. In contrast, overweight and obesity were associated with higher FEV_1_ and even more so with FVC. This likely reflects physical characteristics, as reference values are adjusted for height [28] but individuals of the same height may have different constitutions. In the context of lung function, this is of importance for intrathoracic volume. Further, weight does not differentiate varying proportions of fat and muscle mass. Higher frequency of physical activity was also associated with increased FEV_1_ and FVC. The positive effects found for overweight was weaker or absent for obesity, suggesting that further weight increase beyond overweight is not beneficial. Notably, reduced FEV_1_/FVC, the hallmark of airway obstruction, was found for overweight and more so for obesity as compared to normal weight. These findings might be explained by airway dysanapsis, resulting from a proportionally larger growth of the lung parenchyma in relation to airways in childhood, negatively affecting lung geometry and respiratory parameters. Clinically, such a picture is associated with increased severity among those with asthma [40].

Importantly, associations found in cross‐sectional assessments should be interpreted with caution, as the temporal sequence of exposures outcomes are unknown, and the presence of simultaneous asthma might modify the association [9]. However, in population‐based cohorts, low BMI during childhood has been linked to impaired lung function in adulthood [2, 41].

### 4.4 Strengths and Limitations

The longitudinal design and the large population‐based sample with high participation supports the validity of our results. Lung function and allergic sensitization were assessed with objective and standardized methods, and the SPTs have been repeated and validated against serum IgE with good agreement [17]. Mean FEV_1_ and FVC in the study sample was slightly lower than GLI reference values which is expected as our study population was unselected and thus also included non‐healthy individuals, such as those with asthma. A limitation was that the number of asthmatics did not allow more detailed phenotyping of asthma and deepened analyses of their association with lung function.

## 5 Conclusion

Among schoolchildren followed from 8 to 19 years, aeroallergen sensitization was not independently associated with lung function, despite being an important factor in asthma. This applied irrespective of age at onset, type or level of sensitization. Early‐onset asthma was indeed associated with impaired lung function at 19 years and of similar magnitude among sensitized and non‐sensitized individuals. Associations between foetal and early life factors and lung function underlines the significance of this period for lifelong health.

## Author Contributions

E.R. designed and founded the longitudinal cohort study. E.R., J.B., and L.H. contributed to the design of the current study. J.B. and E.R. did the statistical analysis and verified the underlying data. J.B. and E.R. drafted the report. E.R., L.H., A.B. and M.P. collected data. All authors contributed to data interpretation, provided important content, and reviewed the final report.

## Conflicts of Interest

The authors declare no conflicts of interest.

## Supporting information

Supporting Information S1

Supporting Information S2

## Data Availability

The datasets used and/or analysed during the current study are available from the corresponding author on reasonable request.
