# Supplementary material for: Early Onset Asthma, But Not Aeroallergen Sensitization, Is Associated With Lung Function Impairment in Young Adulthood—A Prospective Cohort Study
Source: Clin Transl Allergy. 2025 Jul 23;15(7):e70084. doi: 10.1002/clt2.70084 (PMC12286707; doi:10.1002/clt2.70084)
Supplement: Supplementary file 1 — Supporting Information S1 [file CLT2-15-e70084-s001.docx]

**Online supplement**

Table S1. Definitions of variables and questionnaire items

| **Definition** | **Question in the questionnaire completed by the parents/teenager.** |
| --- | --- |
| ***Questionnaire responses at age 8, 12 and 19 years*** | |
| Physician diagnosed asthma | Has your child/you been diagnosed by a physician as having asthma? |
| Wheeze last 12 months | Has your child/you had wheezing or whistling in the chest in the last 12 months? |
| Asthma medication last 12 months | How often has your child/you had to use asthma medication in the last 12 months (sometimes, often/periodically, or everyday)? |
| Current asthma | Physician diagnosed asthma and either wheeze or use of asthma medication during the last 12 months. |
| ***Questionnaire responses at age 8*** |  |
| Parental asthma | Is there asthma in mother or father? |
| Maternal smoking in pregnancy | Did the child’s mother smoke during the pregnancy? |
| Birthweight <2500 grams | What was your child’s birth weight? |
| Breastfeeding <3 months | Until what age was your child fed breast milk? |
| Severe respiratory infection | Has your child had whooping cough, croup, pneumonia or severe airway disease, e.g. RS virus? |
| Traffic pollution exposure | A large busy road or a frequented bus stop within 200 meters of the home? (Yes to current or previous residence) |
| ***Questionnaire responses at age 19*** |  |
| Current allergic rhinitis | Physician diagnosed asthma and either symptoms of rhinitis or use of medication for allergic rhinitis during the last 12 months. |
| Smoking | Do you smoke at least 1 cigarette daily or almost daily? |
| Exercise at least 4 times/week | How often do you exercise (answer at least 4 times/week or more) |
| BMI | BMI was based on height and weight measurements and categorized according to WHO criteria as Underweight (<18.5) Normal (18.5-24.9), Overweight (25-29.9) and Obesity (≥30). |

Table S2. Prevalence (%) of risk factors for lung function in the study sample, by sex and in total.

|  |  | Females | |  | Males | |  | All | |  | P-value, difference by sex* |
| --- | --- | --- | --- | --- | --- | --- | --- | --- | --- | --- | --- |
| Risk factors |  | n=747 | (%) |  | n= 748 | (%) |  | n= 1495 | (%) |  |  |
| ***Factors assessed at age 8 years or longitudinally*** | |  |  |  |  |  |  |  |  |  |  |
| Age at onset of sensitization | Never | 441 | (59.0) |  | 394 | (52.7) |  | 835 | (55.9) |  | 0.013 |
|  | 12-19 years | 97 | (13.0) |  | 87 | (11.6) |  | 184 | 12.3) |  | 0.425 |
|  | 8-12 years | 68 | (9.1) |  | 92 | (12.3) |  | 160 | 10.7) |  | 0.046 |
|  | <8 years | 141 | (18.9) |  | 175 | (23.4) |  | 316 | 21.1) |  | 0.032 |
|  |  |  |  |  |  |  |  |  |  |  |  |
| Number of positive SPT at 8 years | 0 | 606 | (81.1) |  | 573 | (76.6) |  | 1179 | 78.9) |  | 0.032 |
|  | 1 | 64 | (8.6) |  | 72 | (9.6) |  | 136 | 9.1) |  | 0.477 |
|  | 2 | 36 | (4.8) |  | 40 | (5.3) |  | 76 | 5.1) |  | 0.642 |
|  | 3 or more | 41 | (5.5) |  | 63 | (8.4) |  | 104 | 7.0) |  | 0.026 |
|  |  |  |  |  |  |  |  |  |  |  |  |
| Asthma category | Never | 646 | (86.5) |  | 642 | (85.8) |  | 1288 | (86.2) |  | 0.716 |
|  | Late-onset | 68 | (9.1) |  | 54 | (7.2) |  | 122 | (8.2) |  | 0.183 |
|  | Early-onset-remission | 9 | (1.2) |  | 25 | (3.3) |  | 34 | (2.3) |  | 0.006 |
|  | Early-onset-persistent | 24 | (3.2) |  | 27 | (3.6) |  | 51 | (3.4) |  | 0.673 |
|  |  |  |  |  |  |  |  |  |  |  |  |
| Birthweight | <2500 g | 28 | (3.9) |  | 27 | (3.8) |  | 55 | (3.8) |  | 0.915 |
| Severe respiratory infection | Yes | 423 | (56.6) |  | 425 | (56.8) |  | 848 | (56.7) |  | 0.940 |
| Maternal smoking during pregnancy | Yes | 176 | (23.8) |  | 164 | (22.3) |  | 340 | (23.0) |  | 0.493 |
| Parental asthma | Yes | 117 | (15.7) |  | 147 | (19.7) |  | 264 | (17.7) |  | 0.043 |
| Breastfeeding < 3 months | Yes | 113 | (15.5) |  | 94 | (12.9) |  | 207 | (13.8) |  | 0.154 |
| Traffic pollution exposure | Yes | 317 | (42.4) |  | 304 | (40.6) |  | 621 | (41.5) |  | 0.481 |
|  |  |  |  |  |  |  |  |  |  |  |  |
| ***Factors assessed at 19 years*** |  |  |  |  |  |  |  |  |  |  |  |
| Sensitization to any animal | Yes | 224 | (30.0) |  | 282 | (37.7) |  | 506 | (44.8) |  | 0.002 |
| Sensitization to any pollen | Yes | 234 | (31.3) |  | 283 | (37.8) |  | 517 | (34.6) |  | 0.008 |
| Number of positive SPT at 19 years | 0 | 452 | (60.5) |  | 401 | (53.6) |  | 853 | (57.1) |  | 0.007 |
|  | 1 | 76 | (10.2) |  | 71 | (9.5) |  | 147 | (9.8) |  | 0.658 |
|  | 2 | 51 | (6.8) |  | 50 | (6.7) |  | 101 | (6.8) |  | 0.912 |
|  | 3 or more | 168 | (22.5) |  | 226 | (30.2) |  | 394 | (26.4) |  | <0.001 |
|  |  |  |  |  |  |  |  |  |  |  |  |
| Current allergic rhinitis | Yes | 102 | (13.7) |  | 106 | (14.2) |  | 208 | (13.9) |  | 0.823 |
| Smoker | Yes | 84 | (11.2) |  | 61 | (8.2) |  | 145 | (9.7) |  | 0.044 |
| Excercise ≥ 4 times/week | Yes | 255 | (34.1) |  | 328 | (43.9) |  | 583 | (39.0) |  | <0.001 |
| BMI categories** | Underweight | 72 | (9.6) |  | 31 | (4.1) |  | 103 | (6.9) |  | <0.001 |
|  | Normal | 553 | (74.0) |  | 457 | (61.1) |  | 1010 | (67.6) |  | <0.001 |
|  | Overweight | 83 | (11.1) |  | 194 | (25.9) |  | 277 | (18.5) |  | <0.001 |
|  | Obesity | 38 | (5.1) |  | 66 | (8.8) |  | 104 | (7.0) |  | <0.001 |
| * Chi square test for proportions, t-test for numerical variables. | | |  |  |  |  |  |  |  |  |  |

**Underweight=BMI<18.5, normal=BMI 18.5-24.9; overweight=BMI 25-29.9, obesity=BMI>30

Table S3. FEV_1_ and FVC presented in percent of predicted (% pred) and standard deviation (SD), and FEV1/FVC (SD) at age 19 years by risk factors.

| Risk factors | FEV_1_, % pred (SD) | p-value* | FVC, % pred (SD) | p-value* | FEV_1_/FVC (SD) | p-value* |
| --- | --- | --- | --- | --- | --- | --- |
| ***Assessed longitudinally or at age 8 years*** | |  |  |  |  |  |
| Age at onset of sensitization |  |  |  |  |  |  |
| Never | 97.9 (10.3) | 0.179 | 96.5 (10.7) | 0.153 | 0.88 (0.06) | 0.267 |
| 12-19 years | 97.0 (12.3) |  | 95.4 (11.4) |  | 0.88 (0.06) |  |
| 8-12 years | 99.3 (10.0) |  | 98.1 (10.8) |  | 0.87 (0.06) |  |
| <8 years | 97.4 (10.4) |  | 96.6 (10.5) |  | 0.87 (0.06) |  |
| Asthma category |  |  |  |  |  |  |
| Never | 98.1 (10.3) | **0.001** | 96.5 (10.7) | **0.021** | 0.88 (0.06) | **<0.001** |
| Late-onset | 97.6 (12.1) |  | 97.4 (11.6) |  | 0.87 (0.06) |  |
| Early-onset-remission | 92.6 (10.3) |  | 92.0 (10.7) |  | 0.87 (0.05) |  |
| Early-onset-persistent | 94.2 (11.4) |  | 99.1 (10.3) |  | 0.82 (0.07) |  |
| Birthweight <2500 g |  |  |  |  |  |  |
| No | 97.9 (10.4) | 0.223 | 96.6 (10.7) | 0.381 | 0.88 (0.06) | 0.967 |
| Yes | 96.1 (11.5) |  | 95.3 (12.8) |  | 0.88 (0.07) |  |
| Respiratory infection |  |  |  |  |  |  |
| No | 98.4 (10.3) | 0.054 | 96.8 (10.8) | 0.536 | 0.88 (0.06) | **0.028** |
| Yes | 97.4 (10.7) |  | 96.4 (10.7) |  | 0.87 (0.06) |  |
| Maternal smoking |  |  |  |  |  |  |
| No | 98.0 (10.7) | 0.383 | 96.3 (10.9) | 0.071 | 0.88 (0.06) | **<0.001** |
| Yes | 97.4 (9.93) |  | 97.5 (10.1) |  | 0.87 (0.06) |  |
| Parental asthma |  |  |  |  |  |  |
| No | 97.9 (10.5) | 0.301 | 96.5 (10.8) | 0.488 | 0.88 (0.06) | **0.002** |
| Yes | 97.2 (10.6) |  | 97.0 (10.5) |  | 0.87 (0.06) |  |
| Breastfeeding < 3 months |  |  |  |  |  |  |
| No | 97.9 (10.5) | 0.300 | 96.6 ± 10.7 | 0.536 | 0.88 ± 0.06 | 0.807 |
| Yes | 97.1 (10.8) |  | 96.1 ± 10.7 |  | 0.88 ± 0.06 |  |
|  |  |  |  |  |  |  |
| Traffic pollution exposure |  |  |  |  |  |  |
| No | 97.9 (10.7) | 0.734 | 96.7 ± 10.9 | 0.521 | 0.88 (0.06) | 0.573 |
| Yes | 97.7 (10.3) |  | 96.4 ± 10.6 |  | 0.88 (0.06) |  |
|  |  |  |  |  |  |  |
| ***Factors assessed at 19 years*** | |  |  |  |  |  |
| Sensitization to any animal |  |  |  |  |  |  |
| No | 98.0 (10.4) | 0.263 | 96.6 ± 10.9 | 0.996 | 0.88 (0.06) | **0.008** |
| Yes | 97.4 (10.8) |  | 96.6 ± 10.6 |  | 0.87 (0.06) |  |
| Sensitization to any pollen |  |  |  |  |  |  |
| No | 97.8 (10.4) | 0.884 | 96.5 ± 10.7 | 0.793 | 0.88 (0.06) | 0.356 |
| Yes | 97.9 (10.8) |  | 96.7 ± 10.8 |  | 0.88 (0.06) |  |
| Number of positive SPT |  |  |  |  |  |  |
| 0 | 97.9 (10.3) | 0.696 | 96.5 ± 10.7 | 0.619 | 0.88 (0.06) | 0.258 |
| 1 | 97.2 (11.8) |  | 95.9 ± 11.6 |  | 0.88 (0.05) |  |
| 2 | 97.0 (11.2) |  | 96.2 ± 11.9 |  | 0.87 (0.06) |  |
| 3 or more | 98.1 (10.4) |  | 97.1 ± 10.3 |  | 0.87 (0.06) |  |
| Current allergic rhinitis |  |  |  |  |  |  |
| No | 97.7 (10.6) | 0.350 | 96.4 (10.9) | 0.155 | 0.88 (0.06) | 0.327 |
| Yes | 98.5 (10.2) |  | 97.6 (9.9) |  | 0.87 (0.06) |  |
| Smoker |  |  |  |  |  |  |
|  |  |  |  |  |  |  |
| No | 98.0 (10.5) | 0.137 | 98.0 (10.5) | 0.171 | 0.88 (0.06) | 0.884 |
| Yes | 96.6 (11.0) |  | 96.6 (11.0) |  | 0.88 (0.05) |  |
| Exercise ≥ 4 times/week |  |  |  |  |  |  |
| No | 96.9 (10.7) | **<0.001** | 95.8 ± 10.8 | **<0.001** | 0.88 (0.06) | 0.572 |
| Yes | 99.2 (10.3) |  | 97.8 ± 10.7 |  | 0.88 (0.06) |  |
| BMI category** |  |  |  |  |  |  |
| Underweight | 91.4 (10.5) | **<0.001** | 87.9 (10.6) | **<0.001** | 0.91 (0.06) | **<0.001** |
| Normal | 97.9 (10.3) |  | 96.2 (10.2) |  | 0.88 (0.06) |  |
| Overweight | 99.6 (9.9) |  | 99.9 (10.5) |  | 0.86 (0.05) |  |
| Obesity | 98.3 (12.0) |  | 99.6 (11.7) |  | 0.85 (0.05) |  |
| *Independent samples t-test for comparisons between 2 groups, ANOVA for groups with more than 2 groups  **Underweight=BMI<18.5, normal=BMI 18.5-24.9; overweight=BMI 25-29.9, obesity=BMI>30 | | | | | |  |
